# The Nursing Supplement

**Published:** 1888-06-16

**Authors:** 


### The Nursing Mirror.

All Contributions for this Supplement should be addressed "The Nursing Mirror," care of The Editor, 38, Old Jewry, E.C.

### En Passant.

THE SWANSEA MEMORIAL.—Gallant little Wales has no idea of being left out of the jubilee nursing scheme, and has petitioned that Swansea be made the nursing centre for that country. Since Ireland and Scotland are to have centres there is no reason why Wales should not have one too, and the most suitable spot would be Swansea. It is in the middle of a large mining, manufacturing, and agricultural population, and has an excellent hospital containing 130 beds, where nurses are thoroughly trained. Lord Jersey, Sir Hussey Vivian, and others have signed the petition, so it is to be hoped it will secure the attention it merits, and that the Women's Jubilee Fund will be found equal to aiding district nursing in Wales.

STAFFORDSHIRE INSTITUTION FOR NURSES.—With the Bishop of Shrewsbury for chairman and treasurer, this institution at Stoke-upon-Trent, is doing good work. It has supplied six district nurses to neighbouring villages, and it nurses gratuitously, or on reduced terms, those who ars too poor to pay the full fees. The Queen has presented the institution with two volumes of her "Leaves from the Highlands," with an autograph inscription. In the balance-sheet there is one item which strikes us with dismay: levied in fines from nurses during last year was the sum of £10. Surely discipline might be maintained without such extortion as this.

THE PROVINCES AND THE PENSION FUND.—At a meeting of the nurses on the subject of the Pension Fund, held at Gloucester Infirmary on May 25th, there were present the Senior Physician, the Secretary, several members of the Committee, the Matron, and the nurses. Dr. Batten said: "We are not here to-night to press you to do anything, but simply to explain to you the advantages of this Society. We do not consider as men that we have a right to go on without making some provision for a rainy day, and I should not liked to have got married unless I could have made some provision for my wife and children. I think some of you really try to save, but it sometimes happens that a friend comes and asks you to lend her a sovereign, and you being so kind-hearted cannot say no, and you never get it again. Then as soon as you have a little money saved up there is a new dress which you must have, and at the end of the year there is nothing saved. Now, if you put your money into the National Pension Fund you cannot possibly spend it in a new dress and it will be saved up for a rainy day. I am sure you will never regret placing your money in this National Pension Fund, and if you once begin paying you will not care to discontinue it, and it will be putting your money into a safe box, of which you have not got the key. Mr. Pike will, I am sure, deduct the necessary amount each month, if you wish it, from your salary, and when you come to the age of 50 or 60 there will be a nice little fortune for you, and if you ever get married or decide to give up your vocation you can, of course, withdraw all your contributions or still continue paying in." Dr. Ancrum also spoke, and Mr. Whitcombe, who has shown the deepest and most intelligent interest in the Fund, read a valuable paper explaining the working of the Fund and the advantages it offers, which may be had on application to him at the Infirmary, Gloucester. The Matron (Miss Yeats), set a good example by joining the Fund, which is being followed by increasing numbers every week.

A WOMANLY QUEEN.—The current number of the *National Review* contains an article on the Queen of Roumania in which some charming pictures are given of an earnest and beautiful life. During the Russo-Turkish war the Queen worked personally amongst the wounded; she assisted at operations, helping to administer chloroform, and she dressed the wounds of her soldier-subjects with her own hands. Ever since that time her people have called her "Muma ranitilor," the mother of the wounded.

CHEAP NURSING LECTURES.—The great success of the Polytechnic as an institute for young men has led to the securing of premises nearly opposite for an institute for young women. Here are class-rooms, reading-rooms, and social parlours where business girls can spend pleasant evenings; and for the moderate fee of sixpence any member can attend a class of ambulance and nursing lectures of a practical nature.

DISTRICT NURSING IN BELFAST.—On the 23rd of last month the Belfast Society for Providing Nurses for the Sick Poor held its annual meeting, and speeches were made by the Mayor, the Rev. Dr. Hannay, the Rev. Dr. Murphy, and others. An extra nurse, to relieve the other nurses when pressure was great in any particular district, had been provided by the generosity of Mr. M'Niele, who promised £130 a-year for ten years for this purpose. The same gentleman had also sent a cheque to supply new air cushions and water beds, which were greatly needed. The plan originated by Miss Philippa Hicks, now matron of the Hospital for Sick Children, Great Ormond Street, of getting ladies in the country to invite nurses to be their guests had been successfully adopted, and two nurses had thus enjoyed a well-merited holiday. During last year the nurses attended over 800 patients, and satisfactorily carried out the work of the society.

THANKS TO THE MATRON.—The last paragraph of the report of the Staffordshire Institution runs as follows: "The committee know well how much they are indebted for the success which they have from year to year been able to record, to the admirable management, constant watchfulness, and experienced judgment of their lady superintendent, Miss Shirley. In tendering to her their warmest thanks for the patient labours of another year, they hope that she may be long spared to preside over an institution in whose prosperity she is so deeply interested, and whose continued good name must be to her a cause of such true satisfaction."

THE GLASGOW TRAINING HOME.—This is a home with a private hospital attached, where nurses are trained. There are nearly a hundred nurses belonging to the institution, and last year they nursed 216 cases in the hospital and 448 cases of sickness in private families. Probationers wishing to join the home must be over 24 years of age, and if found suitable after a month's trial, must bind themselves to serve the institution for three and a-half years at a salary rising from £14 to £20. The home was started in 1874, and at first worked under great difficulties, but is now self-supporting. If the finances of the institution continue to flourish, it is proposed to build a cottage home of rest for the nurses in some pretty country spot. There are some lovely villages down the Clyde, easily got at both by steamer and rail now, where nurses could enjoy absolute quiet and sea air in the midst of the most beautiful scenery. Their salaries are so small, it is only right the institution should secure them pleasant holidays when they can be spared from work.

### Notes from America.

HOSPITAL LIFE IN AMERICA.—In this month's *Scribner* there is a charming article on the hospitals of New York, and it is illustrated with those beautiful engravings which American magazines produce so much better than we do. We quote the following description of the Nurses :—"It is cheerier yet within the walls. Such a regiment of bright-faced, energetic young women would enliven a dungeon. I used to feel inclined to ask them if attractiveness was one of the requirements in their examination as nurses. But their dignity overruled my hazardous impulses, and I never so much as mentioned the fact that I took in agreeable doses of Miss S——'s eyelashes and Miss G——'s dimples, together with the contents of the glasses held to my lips. The trim figures in the blue and white striped gowns and white aprons, the intelligent faces under the round muslin caps, are comforting sights for a man to open his eyes upon after a bad time. The nurses do not appear to know this. They seem as engrossed in critical cases and capital operations as are the medical students yonder, pouring out of the lecture-room, note-book in hand. I didn't object to the boys, even when they wanted to learn their lessons off my bones, to sound my chest and listen to my bellows. But the young women, with equal zest for information, were more shrewd about it and asked fewer questions. They looked sharp and missed nothing. And the demure airs they gave themselves over their caps and their titles—Junior, Middle, and Senior—their interest in their charges and fondling ways with the children, were an inexhaustible source of entertainment for at least one old fellow who watched. They were justly proud of their clean wards, too, and of their neatly-arranged '*T. I. Ds.*'—the medicine-closets, so-called from the *Ter-in-die* (*thrice-a-day*) doses therein contained."

AMONG the candidates for the Presidency of the United States is Senator Hawley, General U.S.A., to whom for reasons quite other than political, we wish every success. Our interest in Senator Hawley lies in the fact that his wife, formerly Miss Edith Hornor, is a trained nurse, and at the time of her marriage was assisting Miss Alice Fisher, matron of the Philadelphia Hospital. Miss Hornor is an Englishwoman, and was trained at the Radcliffe Infirmary, Oxford. She accompanied Miss Fisher to America three and a-half years ago. Mrs. Hawley is still devoted to her profession, and is doing all she can to give it the place it deserves in public esteem, and if she were mistress of the White House, we do not doubt that nursing would receive an impetus in both thoroughness and dignity of position that would be felt throughout the whole continent.

WE regret to hear that Miss Alice Fisher, the head of the great Nurse-training School at Philadelphia, has been suffering severely for several months from rheumatism, complicated by heart disease, and is still in a very weak state of health. Her place is meanwhile being filled by her assistants, Miss Anne Murray, formerly of the Royal Infirmary, Edinburgh, and Miss Roberta West, the first pupil entered on the books of the school, and gold medallist in all her classes. At a meeting of women held in Philadelphia on May 14, it was unanimously resolved that as a token of appreciation of Miss Fisher's faithful and efficient care for the suffering poor of the community, a suitable building should be erected as soon as practicable in the immediate neighbourhood of the Philadelphia Hospital, for the accommodation of its nurses and the graduates of its training school, the building to be called the "Alice Fisher Home for Nurses." A more fitting and desirable recognition of Miss Fisher's services could not be imagined, nor one that would please her more.

### Original Articles.

### OLD AND NEW NURSES.

#### Both Sides of a Burning Question.

WE have received a letter from an old nurse of fifteen years experience, which almost reads like a reply to Miss Twining's letter published in this month's *Work and Leisure*. It is always very hard to see both sides of a question, and for a trained nurse to recognise that good work can be done by her humbler uncertificated sister requires a breadth of mind and an unprejudiced judgment not often found. The hospital nurse is apt to pin her faith to a certificate, and to look down on those who, having taken up nursing before the days of examinations, have only experience on their side. The following are the letters referred to :—

#### Miss Twining's Opinion.

"As a supporter of your magazine from the beginning, I venture to express my regret at the encouragement that has been given by one of the recent articles to the idea that anybody, trained or not, is fit and able to manage a hospital and its patients. Efforts are being made now, by all who understand the subject, in exactly the contrary direction, and it is in order to check the serious evils that arise from incompetency, that a scheme of registration for all nurses is being set on foot and earnestly advocated. When we find women who have been *six weeks* in some kind of institution being called, and sent out as, 'Nurses,' I think it is time to protest against all such fallacies, and to urge that amateurs should not be allowed to take the place and work of those who have gone through an efficient training, and have obtained certificates."

#### "An Old Nurse's Thoughts as she went about District Nursing.

"Being a reader of The Hospital, *Nursing Notes*, and another nurses' paper, I think there is a great deal more written and talked about nurses being highly trained than is necessary. I suppose the training schools do train their nurses sufficiently, if not, it is a pity. Private and district nurses do not require the same amount of training, or else how is it that the old nurses, whom some would call now only partly trained, have for years back been able to give satisfaction, obtain satisfactory and excellent certificates, and that our lady superintendents have heard time after time what a comfort the nurse has been in a family. Now what more was required, or could have been done by the most highly trained that are to be? The more highly-trained the nurse is, the more difficult will she find it to stand by as if she were merely an uneducated domestic servant, which is often required of a nurse in a private family. It is the great amount of tact, humility, patience, and cheerfulness, that is required of a private nurse, as well as a certain amount of hospital training. Hospitals are not training schools for these first three virtues. What use would the nurse of three years' training be in a private family, when it is these things that are so much wanted? In many cases a private nurse is expected to know, but to act as if she knew nothing. What is high hospital training in these cases? The probability is that the highly-trained nurse would be sent home in haste. In private surgical cases the doctor mostly does the dressings himself for the first few times, but after all we need not trouble, for I think we shall continue to be useful, and to be a comfort to many more families to the end, and let the fresh ones go in for more training if it is necessary. It certainly will be a good thing if more of the poor can be nursed by even partly-trained nurses. I can remember the time when many families would have been glad to get partly-trained nurses, so I flatter myself we are a great improvement upon the past. The most highly-trained nurse of the future can only say the same of us when we are gone that we say of those that are already gone. I take this opportunity of thanking you for taking so much trouble and giving so much of your time for the benefit of nurses, as you have done about the National Pension Fund. Whether it is a success or not, many, many thanks are due to the founder of it. If you think these few thoughts are worth inserting, please do so, and accept my apology for taking up your valuable time."

Surely it is a very rare thing to find that women of only six weeks' training are sent out as competent nurses? It is well known that some of the institutions for private nurses are not careful enough in their choice of members; but the public can always avoid the doubtful nurse by going to a nursing institution connected with one of the large hospitals. Miss Twining, who knows so well what our paupers have suffered at the hands of the race of Sarah Gamps, must also have seen the good work done by many district nurses, who have never been through an examination in their lives. As our correspondent says, tact, humility, patience, and cheerfulness are as necessary as hospital training. With the new race of nurses which has grown up in these latter days there have sprung up new temptations of a more subtle character, difficult to see and therefore hard to conquer. The lady nurse engaged in district work blacks the grate and goes down on her knees and thinks it is all humility: perhaps it is all pride. Both amongst the poor and the rich doctors must often have heard the request, "Please don't send me a lady nurse." The lady nurse will not drink, and she will administer medicine and applications punctually, but in a thousand ways she may irritate and annoy her patient. The unbending habits of a hospital should be slightly relaxed in private life, and domineering ways should be avoided. A lady who was for four years a sister in a hospital, and who married a doctor, when her time of trial drew near, begged to have the services of some motherly old woman of the servant class; she thought she would be so much more easy to deal with, and give less trouble, than a certificated nurse. There can be no doubt that it is a good thing that in nursing, as in other work, the old order has changed giving place to new; but it is as well to insist that old faults have given place to new also. In her pride the nurse of to-day may be far too ready to forget the Christian virtues and sweet womanly ways which her knowledge should adorn, not disfigure. It is so hard to hit the happy medium, and though well trained and strong of nerve, not to degenerate into a mere clever nurse and an unsympathetic woman. The matrons of most of our hospitals acknowledge that those who are first in the examinations are not always the best nurses. We have set up a fetish called a certificate, and decided to bow down and worship it, not insisting sufficiently that it is mere dry parchment to the holder, unless she combines with it the living beauty of an unselfish and godly character. To disparage the trained nurse in pages meant especially for her perusal seems an unkind deed, but none can desire thorough training for every nurse more than ourselves. Only we would ask those who have mixed much in modern hospital life if they have not come across specimens of women who have deteriorated through their eager race for mere knowledge, which left them no time to remember the healing virtues which—

"Lie scattered at the feet of men like flowers."

### Useful Hints.

[Contributions for this Column are invited.]

Bran Fomentations.—After wringing the flannel containing the bran out of hot water, have a large pot simmering by the fire with a little water in it, and bowl standing in it. Put the bag in the pot, resting on the bowl, so as not to touch the water. The steam will heat the bran thoroughly without the necessity of wringing again out of hot water. There should be two bags, and while one is applied to the patient the other is kept in the pot.

To Dress Ulcers.—Wash the surrounding skin and the surface of the ulcer with sublimate lotion 1·1000. Cut strips of isinglass plaster long enough to encircle the limb. Pass the strips through a hot solution of carbolic acid about 1·100, and apply in the usual way. Bandage the limb. Do not remove the dressing unless there is an offensive smell.

### A District Matron's Diary.

### A WEEK'S WORK.

We are again able to lay before our readers further pages from the diary of Miss Taylor, matron of the East London Nursing Society :—

*March* 23*rd*.—Three new serious cases. Stow, suffering from rheumatic gout, was removed to the hospital two days after we took up the case, needing more help than a district nurse could give. Baby Boys suffering from measles and bronchitis; he is kept very warm, medicine is given, and poultices applied. Marks, a man suffering from lung mischief, is glad to have his bed made; malt extract is given, also nourishment from the vicarage. Grant, who has been suffering from dropsy, is better; the others on the register are also improving.

*March* 31*st*.—Four new cases. Peters, suffering from a blow on the head caused by an iron bar falling. He has also a skin eruption, which I think comes from cold. We are dressing the wound with ointment, and using lead lotion, to reduce the inflammation. Mrs. W., suffering from ulceration of the leg; we cleanse it daily, and dress it with zinc ointment. Mrs. Lot, a poor woman, very ill with heart disease, her brain also affected; we have persuaded her to go to the infirmary, and I hear since, she has been removed to an asylum. The last new case is a woman on whose head a shutter fell; her eyes are very black, and she is rather weak, but will soon be all right. Baby Boys has recovered. Poor Grant has been tapped to-day.

*April* 9*th*.—One new serious case. Patsy, suffering from a badly-lacerated foot, which nurse dresses daily. He is an out-patient at the hospital. I am glad to say that since Grant was tapped the water has been dispersed by medicine and he is steadily improving. He is very hopeful, which is a great help towards recovery, in all cases. Peters has recovered from the blow on the head, but is still troubled by the skin disease. Mrs. W. says she blesses the day nurse found her out and began to dress her legs; they are so much better now they are thoroughly attended to. The chronic cases are much as usual.

*April* 15*th*.—No new serious cases. The man Patsy, with a lacerated foot, is getting all right again. Poor Grant is allowed to get up for a short time every day, and is more hopeful than ever. Peters, who was recovering from skin disease, has had a return of it; I am sorry, for he has a wife and five children to support, and will not be able to work for some time. Mrs. W. is about the same; I see no chance of her legs ever getting quite well. The rest are chronic cases, all very grateful for a visit and needing occasional help. Some are suffering from rheumatism, others from phthisis, bronchitis, etc.

*April* 23*rd*.—No new serious cases. Mrs. W. is very thankful for our care; sad to say her husband, who had been out of work for weeks, has met with an accident. While employed the first day in pulling down an old house, he fell from the roof, contracting serious injuries. He was taken to the hospital, and though out of danger now, will never be able to work again. Patsy's foot is quite well, and he can come off the books. Grant looks very happy sitting up in an easy-chair; I am glad to say there has been no return of the dropsy since he was tapped.

*April* 30*th*.—Three new serious cases. Tommy, a child, suffering from croup. Nurse has made a beautiful little tent and arranged a steam kettle splendidly. I think the child will recover, for nurse, for a wonder, was sent for in time. Jones, suffering from chronic asthma. He is very thin, and sore from lying in bed so long; we have supplied him with a water pillow. Mrs. Mitchell has had a bad confinement and sore breasts. Nurse is most clever and kind to her, and I hope she will do well.

### Everybody's Opinion.

### THE PATIENT SPEAKS.

You have all sorts writing to you it seems to me except the patients, and, after all, you couldn't get on without us. We see The Hospital when the nurses chuck it aside, and we think we don't get enough notice. We have our opinion of nurses, and that is, that they are often hard and careless of our feelings. Men don't like to show when they're feeling bad and make a fuss, but still they like sympathy. Nurses do get so used to sad sights and sounds that they forget that they are sad. When I hear a man groaning it's worse than my own pain, but the nurses don't seem to heed. They are attentive enough, and kind and gentle too in their way, but I shan't pick a wife out of those who have been waiting on me. Woman should be real womanly, and as soft and tender as young asparagus. James Macfarlane.

[Our correspondent's views are not those of many patients. We should be glad to have letters from any patients who have something to say worth reading or of general interest.—Ed. *T.H.*]

### PRESENCE OF MIND v. PANIC.

With reference to the experience of "Aidyl" in last week's Hospital, I am afraid her account of a night adventure would not be calculated to inspire invalids with confidence, or impress the public with the courage or efficiency of trained nurses. In all directions to those training for the profession, calmness and self-control are insisted on as necessary qualifications. With a sufficiently serious case to require a day and night attendant, would a nurse be justified (or could she be excused) for screaming so that she roused the household? at an unexplained noise in the night, which proved, after all, to be only the rattling of the front door chain, where the presence of a policeman seems to point to a house within reach of help. Also, would two nurses be fulfilling their duty who could wait three or four hours upstairs without even trying to ascertain the cause of disturbing sounds, even when, as is asserted, "all was quiet"? Would not such piercing screams be detrimental to an ordinary patient, and even cause death in severe cases? It seems to me that a nurse should be too thoroughly ashamed of such gross and negligent weakness and cowardice to risk injuring her professional sisters by the recital of it. I should certainly consider it would most effectually disqualify "Aidyl" for the serious responsibility of a nurse's post. We will hope that there are few of our noble-spirited self-sacrificing nurses who would leave a patient to the chance of a visit from supposed burglars for three or four hours without at least taking some reasonable precautionary measures for their safety and comfort. I wonder what "Aidyl" would have done in our position during the past winter in a position far more trying to weak nerves than that she describes. At the beginning of the winter I came to take charge of this large building of one hundred rooms, and to hurry forward preparations for the reception of patients. At first I had only the young servant with me, and we slept in the house alone for some time in so unfinished a state that many of the outside doors were without fastenings, and we are not within calling distance of any other house. In three months I added a nurse and another servant to my household, and it was only after some weeks that we had a porter—now the only man in the house. We have frequently been aroused by strange noises in the new flues and pipes, and had to make a round of investigation during the night to ascertain if all were right. Mine has been a much happier experience of nurses. Only a fortnight ago a nurse, who is a most delicate woman, was awakened by a crash in the night caused by the bursting of a large cistern, holding 600 gallons of water. She came quietly into my room, called me without waking my little girl, who was sleeping in my room, or any of the household; and it was not until we had really ascertained from whence the noise proceeded and the extent of the damage, that we attempted to call the porter, who is the only man in the house. A Matron.

### Appointments.

[It is requested that successful candidates will send a copy of their applications and testimonials, with date of election, to The Editor, The Lodge, Porchester Square, W.]

Miss Stevenson has been appointed matron of the Bradford Children's Hospital. Miss Stevenson was trained at the Glasgow Royal Infirmary, and has lately been working in the infirmary at Bristol. We congratulate her upon her success, and hope she may find her new work congenial and pleasant in every way.

### Vacancies.

The following vacancies are announced :—

**Nurses.**

Kidderminster Infirmary; £25,

Bristol Infirmary.

National Hospital, Queen-square; £16.

Southport Infirmary; £20.

Kingston Home, Hull.

Poorhouse, Irvine; £25.

Nottingham Nursing Institution.

Burton-on-Trent Nursing Institution.

Burton-on-Trent Infirmary; £25.

Metropolitan Hospital; £20.

Brighton Fever Hospital; £25.

Nursing Institute, Ryde, I.W.

**District Nurses.**

Grimsby; £80.
Glasgow.

**Probationers.**

Bristol Children's Hospital.
Jenny Lind Hospital, Norwich.
Brighton Fever Hospital.

### Exchange and Mart.

Under this heading we propose to insert purely Exchange advertisements, at a charge of sixpence for eighteen words, or three insertions for one shilling It must be distinctly understood that ordinary advertisements will not be inserted here.

RENSHAWS MANUALS. — "Anatomy," Malgaine (12s. 6d.), "Elementary Botany, Dr. Silver (12s. 6d.), "Human Osteology" Ward (7s.) Would exchange either for Nurse's Surgical dressing case. What offer? Also Owen's "Materia Medica," "A Guide to the New Pharmacopœia" "First Book of Botany, Balfour. The last three for 5s.; or one guinea the lot. All in good condition. Address C., The Hospital Office, 38, Old Jewry, E.C.

